# Supplementary material for: Multi‐dataset identification of innovative feature genes and molecular mechanisms in keratoconus
Source: J Cell Mol Med. 2024 Sep 19;28(18):e70079. doi: 10.1111/jcmm.70079 (PMC11412914; doi:10.1111/jcmm.70079)
Supplement: Supplementary file 2 — Material S2. [file JCMM-28-e70079-s002.zip › 7.GSEA analysis/2.ATOH7/KEGG_MELANOGENESIS.html]

Details for gene set KEGG\_MELANOGENESIS[GSEA]

|  || Dataset | ATOH7.group.cls#h\_versus\_l |
| Phenotype | group.cls#h\_versus\_l |
| Upregulated in class | h |
| GeneSet | KEGG\_MELANOGENESIS |
| Enrichment Score (ES) | 0.44801474 |
| Normalized Enrichment Score (NES) | 1.6807321 |
| Nominal p-value | 0.0 |
| FDR q-value | 0.22350632 |
| FWER p-Value | 0.424 |
Table: GSEA Results Summary

  

Fig 1: Enrichment plot: KEGG\_MELANOGENESIS      
 Profile of the Running ES Score & Positions of GeneSet Members on the Rank Ordered List

  

| SYMBOL | TITLE | RANK IN GENE LIST | RANK METRIC SCORE | RUNNING ES | CORE ENRICHMENT || 1 | TYRP1 | na | 148 | 0.726 | 0.0378 | Yes |
| 2 | WNT1 | na | 172 | 0.697 | 0.0776 | Yes |
| 3 | MITF | na | 236 | 0.652 | 0.1137 | Yes |
| 4 | TYR | na | 333 | 0.610 | 0.1463 | Yes |
| 5 | WNT9A | na | 551 | 0.534 | 0.1711 | Yes |
| 6 | LEF1 | na | 735 | 0.492 | 0.1943 | Yes |
| 7 | WNT6 | na | 825 | 0.474 | 0.2193 | Yes |
| 8 | PRKACB | na | 829 | 0.474 | 0.2467 | Yes |
| 9 | POMC | na | 897 | 0.464 | 0.2717 | Yes |
| 10 | WNT5B | na | 1090 | 0.433 | 0.2913 | Yes |
| 11 | PLCB1 | na | 1718 | 0.367 | 0.2945 | Yes |
| 12 | PRKACG | na | 1784 | 0.361 | 0.3136 | Yes |
| 13 | PRKCB | na | 2281 | 0.324 | 0.3181 | Yes |
| 14 | CAMK2D | na | 2442 | 0.313 | 0.3317 | Yes |
| 15 | FZD7 | na | 2482 | 0.310 | 0.3486 | Yes |
| 16 | DCT | na | 2590 | 0.304 | 0.3631 | Yes |
| 17 | WNT9B | na | 2657 | 0.300 | 0.3786 | Yes |
| 18 | WNT11 | na | 3016 | 0.280 | 0.3845 | Yes |
| 19 | TCF7 | na | 3059 | 0.277 | 0.3994 | Yes |
| 20 | FZD10 | na | 3060 | 0.277 | 0.4155 | Yes |
| 21 | WNT7A | na | 3390 | 0.261 | 0.4211 | Yes |
| 22 | WNT8B | na | 3424 | 0.259 | 0.4352 | Yes |
| 23 | CALML6 | na | 3497 | 0.256 | 0.4480 | Yes |
| 24 | MAP2K1 | na | 4240 | 0.228 | 0.4398 | No |
| 25 | FZD2 | na | 4571 | 0.216 | 0.4428 | No |
| 26 | ADCY3 | na | 5082 | 0.198 | 0.4396 | No |
| 27 | WNT7B | na | 5905 | 0.174 | 0.4259 | No |
| 28 | WNT10A | na | 6547 | 0.156 | 0.4165 | No |
| 29 | TCF7L1 | na | 6644 | 0.153 | 0.4226 | No |
| 30 | EDN1 | na | 7395 | 0.133 | 0.4086 | No |
| 31 | WNT3A | na | 7555 | 0.129 | 0.4115 | No |
| 32 | NRAS | na | 7693 | 0.126 | 0.4149 | No |
| 33 | PRKCA | na | 7938 | 0.120 | 0.4148 | No |
| 34 | FZD4 | na | 8076 | 0.117 | 0.4176 | No |
| 35 | WNT2 | na | 8209 | 0.114 | 0.4205 | No |
| 36 | KITLG | na | 8664 | 0.105 | 0.4135 | No |
| 37 | GNAI1 | na | 9135 | 0.096 | 0.4055 | No |
| 38 | DVL2 | na | 9335 | 0.093 | 0.4051 | No |
| 39 | CREB1 | na | 9769 | 0.085 | 0.3976 | No |
| 40 | CALM3 | na | 10070 | 0.080 | 0.3936 | No |
| 41 | EP300 | na | 10138 | 0.079 | 0.3962 | No |
| 42 | ADCY8 | na | 10448 | 0.075 | 0.3916 | No |
| 43 | WNT4 | na | 10868 | 0.068 | 0.3835 | No |
| 44 | CREB3L2 | na | 11898 | 0.054 | 0.3569 | No |
| 45 | KIT | na | 11952 | 0.053 | 0.3584 | No |
| 46 | FZD8 | na | 11987 | 0.053 | 0.3605 | No |
| 47 | CREB3L4 | na | 12000 | 0.053 | 0.3632 | No |
| 48 | PRKACA | na | 12347 | 0.048 | 0.3560 | No |
| 49 | PRKX | na | 12697 | 0.044 | 0.3484 | No |
| 50 | DVL3 | na | 12971 | 0.041 | 0.3429 | No |
| 51 | DVL1 | na | 13325 | 0.037 | 0.3349 | No |
| 52 | MAPK1 | na | 14536 | 0.025 | 0.3014 | No |
| 53 | HRAS | na | 14559 | 0.025 | 0.3022 | No |
| 54 | GNAS | na | 14632 | 0.024 | 0.3016 | No |
| 55 | WNT8A | na | 15047 | 0.021 | 0.2908 | No |
| 56 | RAF1 | na | 15219 | 0.019 | 0.2870 | No |
| 57 | CREB3 | na | 15750 | 0.015 | 0.2726 | No |
| 58 | EDNRB | na | 15864 | 0.014 | 0.2701 | No |
| 59 | GSK3B | na | 16028 | 0.013 | 0.2661 | No |
| 60 | CALM1 | na | 16251 | 0.011 | 0.2603 | No |
| 61 | FZD6 | na | 16592 | 0.008 | 0.2510 | No |
| 62 | MAPK3 | na | 16821 | 0.006 | 0.2448 | No |
| 63 | CREB3L3 | na | 16998 | 0.005 | 0.2399 | No |
| 64 | CTNNB1 | na | 18214 | -0.006 | 0.2052 | No |
| 65 | CREB3L1 | na | 19199 | -0.015 | 0.1776 | No |
| 66 | GNAI2 | na | 19897 | -0.021 | 0.1587 | No |
| 67 | FZD9 | na | 19998 | -0.022 | 0.1571 | No |
| 68 | WNT3 | na | 20041 | -0.022 | 0.1572 | No |
| 69 | FZD3 | na | 20400 | -0.026 | 0.1483 | No |
| 70 | CALML3 | na | 20596 | -0.028 | 0.1443 | No |
| 71 | GNAQ | na | 20613 | -0.028 | 0.1454 | No |
| 72 | MAP2K2 | na | 20653 | -0.028 | 0.1459 | No |
| 73 | ADCY9 | na | 20701 | -0.029 | 0.1463 | No |
| 74 | PLCB4 | na | 20753 | -0.029 | 0.1465 | No |
| 75 | FZD5 | na | 21276 | -0.035 | 0.1334 | No |
| 76 | ADCY2 | na | 21650 | -0.039 | 0.1249 | No |
| 77 | CALM2 | na | 21949 | -0.042 | 0.1187 | No |
| 78 | TCF7L2 | na | 22134 | -0.044 | 0.1160 | No |
| 79 | CAMK2A | na | 22272 | -0.046 | 0.1147 | No |
| 80 | FZD1 | na | 22280 | -0.046 | 0.1172 | No |
| 81 | GNAO1 | na | 22905 | -0.053 | 0.1023 | No |
| 82 | CAMK2G | na | 23214 | -0.058 | 0.0967 | No |
| 83 | PLCB3 | na | 24121 | -0.071 | 0.0747 | No |
| 84 | WNT5A | na | 24650 | -0.080 | 0.0641 | No |
| 85 | CREBBP | na | 24831 | -0.083 | 0.0637 | No |
| 86 | KRAS | na | 25083 | -0.087 | 0.0614 | No |
| 87 | ADCY5 | na | 25230 | -0.089 | 0.0624 | No |
| 88 | ADCY6 | na | 25322 | -0.091 | 0.0651 | No |
| 89 | ADCY7 | na | 26708 | -0.119 | 0.0320 | No |
| 90 | WNT16 | na | 27162 | -0.130 | 0.0264 | No |
| 91 | WNT10B | na | 27303 | -0.133 | 0.0301 | No |
| 92 | ADCY4 | na | 27391 | -0.136 | 0.0355 | No |
| 93 | GNAI3 | na | 28235 | -0.158 | 0.0203 | No |
| 94 | PLCB2 | na | 28336 | -0.160 | 0.0267 | No |
| 95 | PRKCG | na | 28978 | -0.178 | 0.0185 | No |
| 96 | CALML5 | na | 29931 | -0.208 | 0.0031 | No |
| 97 | ADCY1 | na | 30908 | -0.244 | -0.0109 | No |
| 98 | WNT2B | na | 30926 | -0.244 | 0.0028 | No |
| 99 | ASIP | na | 33394 | -0.404 | -0.0450 | No |
| 100 | MC1R | na | 33756 | -0.449 | -0.0294 | No |
| 101 | CAMK2B | na | 34693 | -0.989 | 0.0010 | No |
Table: GSEA details [plain text format]

  

Fig 2: KEGG\_MELANOGENESIS      
 Blue-Pink O' Gram in the Space of the Analyzed GeneSet

  

Fig 3: KEGG\_MELANOGENESIS: Random ES distribution      
 Gene set null distribution of ES for **KEGG\_MELANOGENESIS**
